# Psychometric properties of the Persian version of short-form five factor borderline inventory (FFBI-SF)

**DOI:** 10.1186/s12888-021-03667-4

**Published:** 2022-02-03

**Authors:** Mojtaba Elhami Athar, Sirvan Karimi, Hilary L. DeShong, Zahra Lashgari, Morteza Azizi, Elham Azamian Jazi, Reza Shamabadi

**Affiliations:** 1 School of Behavioral Sciences and Mental Health (Tehran Institute of Psychiatry), Iran University of Medical Sciences, Tehran, Iran; 2 Shahid Beheshti University of Medical Science, Tehran, Iran; 3 Department of Psychology, Mississippi State University, Starkville, MS 39762 USA; 4 Department of Clinical Psychology, University of Social Welfare and Rehabilitation Sciences, Tehran, Iran; 5 Department of Psychology, Islamic Azad University, Sarab Branch, Sarab, Iran; 6 Ph.D. Student in Educational Psychology, Department of Educational and Counseling Psychology, Faculty of Educational Sciences and Psychology, Ferdowsi University, Mashhad, Iran

**Keywords:** Five-factor model, Borderline personality disorder, Assessment, Maladaptive variants, Persian version

## Abstract

**Background:**

The Five-Factor Borderline Inventory-Short Form (FFBI-SF) is a self-report measure developed to assess traits of Borderline Personality Disorder (BPD) from the perspective of the Five-Factor Model of general personality. This study was designed to examine the factor structure, internal consistency, and convergent/discriminant validity of the Persian FFBI-SF in a sample of Iranian university students.

**Methods:**

A total of 641 university students (M-age = 28.04, *SD* = 8.21, 66.7% women) completed the online forms of the FFBI-SF, PID-5-BF, and Mini IPIP.

**Results:**

Confirmatory factor analysis supported the original and modified (without item 47) twelve-factor models. Also, Cronbach’s alpha (α) for the FFBI-SF scores ranged from unacceptable to excellent ranges. However, when relying on MIC values to measure internal consistency, the FFBI-SF Total and subscale scores demonstrated adequate internal consistency. Finally, the FFBI Total and subscale scores showed the expected relations with other personality measures scores (e.g., Neuroticism, Antagonism, and Conscientiousness), which supports the validity of the interpretation of the FFBI-SF scores.

**Conclusions:**

The findings indicated that FFBI-SF is a useful tool with sound psychometric properties for assessing BPD traits in Iranian students and may spark research in other Iranian settings (e.g., community and clinical samples).

## Background

According to the Diagnostic and Statistical Manual of Mental Disorders, 5th Edition [1], Borderline Personality Disorder (BPD) is “a pervasive pattern of instability of interpersonal relationships, self-image, and affects, and marked impulsivity that begins by early adulthood and is present in a variety of contexts” (p. 663). BPD is diagnosed when individuals meet at least five of the nine diagnostic criteria. Nevertheless, a plethora of studies has indicated the shortcomings of the current DSM diagnostic system, which includes an inadequate scientific basis, arbitrary cutoffs, comorbidity among personality disorders (PDs), comorbidity with other psychological disorders, heterogeneity of diagnoses, and insufficient coverage (e.g., [2–4]).

Another approach to conceptualizing PDs is the dimensional model rather than the categorical one, which has been supported in previous studies (e.g., [2, 5, 6]). In this vein, prior to the publication of the DSM-5, an alternative model for the diagnosis of personality disorders (PD) was proposed [1], which was rejected by the DSM-5 Committee, and the model was published in DSM-5’s section III, i.e., emerging models and measures so that it could gather additional research support and evidence. As such, ongoing studies are providing support for the idea that PDs could be best conceptualized with a dimensional view; more specifically, a growing body of research indicates that BPD is best viewed as a dimensional construct (e.g., [7–10]). Therefore, it may be helpful to assess BPD utilizing dimensional trait measures. Studies on the conceptualization and measurement of personality disorders with the dimensional trait models have supported using the Five-Factor Model (FFM; McCrae & Costa, 2003) to explore the role of personality traits in personality pathology [11]. The FFM comprises five broad domains of personality functioning, including neuroticism, extraversion, openness, agreeableness, and conscientiousness. Further, each of these domains includes six facets [12]. Strong evidence suggests that all ten personality disorders can be conceptualized as maladaptive alternatives of FFM personality traits and that personality disorders represent pathological constellations of fundamental personality traits (e.g., [13, 14]). In this vein, several measures have been developed that assess the maladaptive variants of personality disorders based on the FFM (e.g., [15, 16]). FFM-based measures of personality disorders have a few merits. For instance, such measures allow measuring the maladaptive variants of the FFM, which are not assessed by general FFM measures; thereby, filling the gap between the FFM and the DSM-5 personality disorders by. In addition, dimensional trait approaches like the FFM are more informative than categorical models in that they allow the measurement of more fundamental aspects of PDs [17].

To assess BPD from a dimensional trait perspective, Mullins-Sweatt et al. [18] developed the Five-Factor Borderline Inventory (FFBI). The FFBI was developed based on the empirical evidence suggesting BPD is strongly related to 11 facets of the FFM measured by the Neuroticism-Extraversion-Openness Personality Inventory-Revised [13, 14, 19–22]. Based on these empirical studies, Mullins-Sweatt et al. [18] built 12 subscales that measure the components of BPD. Each dimension is associated directly with a distinct NEO PI-R facet, while the vulnerability facet NEO PI-R contributes to two FFBI subscales. The FFBI subscales include anxious uncertainty (derived from NEO PI-R anxiousness), dysregulated anger (angry hostility), despondence (depressiveness), self-disturbance (self-consciousness), behavioral dysregulation (impulsiveness), affective dysregulation (vulnerability), fragility (vulnerability), dissociative tendencies (fantasy), distrust (trust), manipulativeness (straightforwardness), oppositional (compliance), and rashness (deliberation). In the original study, Mullins-Sweatt et al. [18] developed a measure with 240 items and 20 items for each subscale. The measure was administered with a large undergraduate sample. Then, 120 items were selected based on internal consistency, convergence with the respective NEO PI-R facet scales, and convergence with other BPD scales. The Final version of FFBI includes 120 items (10 items for 12 subscales), which was then validated with a large group of undergraduate students and a clinical sample (i.e., patients with substance use disorder). The results indicated that the FFBI had acceptable internal consistency and was associated well with the NEO PI-R and existing measures of BPD. Furthermore, each FFBI subscale was associated with its corresponding parent NEO PI-R facet. In a second study, DeShong et al. [23] provided further support for the psychometrics (convergent and discriminant validities) of the FFBI by studying two samples of individuals with a history of nonsuicidal self-injury (NSSI). Also, DeShong et al. [23] assessed the associations between FFBI and measures of constructs related to BPD, including impulsivity, emotion dysregulation, early childhood emotional vulnerability, parental invalidation, self-esteem, depression, and anxiety. Thus, the study illustrated further construct validity of the FFBI.

Notwithstanding the benefit of the FFBI, the measure is very long and time-consuming when administered. Therefore, a shorter version of the FFBI may be more beneficial in both clinical and research settings. DeShong et al. [17] developed the short form of the FFBI (FFBI-SF) using item response theory analyses. Their results yielded 48 items, with four items per subscale. The internal consistency of the FFBI-SF subscales ranged from .71 (oppositional) to .86 (affective dysregulation and dissociative tendencies). Also, the FFBI-SF subscales scores were significantly correlated with the full version, which ranged from .85 (dissociative tendencies) to .95 (anxious uncertainty). The FFBI-SF yielded strong convergence with other BPD scales (e.g., MCMI-III) and convergent/discriminant validity with the NEO PI-R scores. Furthermore, Helle et al. [24] indicated that the FFBI-SF predicts specific maladaptive behaviors (e.g., arguing with close family/friends, binging, alcohol misuse, and nonsuicidal self-injury) over time. Beyond this, the FFBI-SF has demonstrated its usefulness in several studies as a dimensional trait measure of BPD, being used in studies related to thought control strategy differences in suicide risk and BPD [25], sleep problems as mediating risk factors for suicide risk within BPD [26], and in studies investigating precursors of the emotional cascade model of BPD [27]. In sum, studies indicate that the FFBI-SF is a valid, informative, and useful measure for assessing BPD from a dimensional trait perspective.

### The current study

While previous studies support the psychometrics of the FFBI-SF, such findings are results of studies from the Western cultures and could not be generalized to Eastern cultures (e.g., Iran) [28–30]. There exist essential variations between Eastern/Asian (e.g., Iran) and Western (e.g., Europe, USA) cultures regarding interpersonal relations, cultural values, and social standards [31], emotional expression [32], and emotional arousal levels [33], which may influence the structure of measures assessing personality in Asian cultures (e.g., Iran) [28–30, 34, 35]. In this regard, different structures of personality constructs due to cultural differences have been indicated in several studies. For instance, the originally proposed five-factor model of the personality inventory for DSM-5 brief form (PID-5-BF; 36) was not replicated with Chinese samples, and a six-factor model was proposed in which the Negative Affect domain was divided into two factors. The new factor labeled “Interpersonal Relationships” was consistent with the Big-Six Personality model in China and reflected the humanistic ethic spirit of Chinese culture [36–38]. Similarly, the FFM did not reach a well-fit model in some Asian countries (e.g., [39–41]), and the Openness dimension of the NEO Personality Inventory was poorly replicated in a study with 24 different Asian cultures, including Iran [42]. Therefore, since the FFBI and its short form have been developed through the lens of FFM, the FFBI-SF might not yield the same factor structure suggested in previous studies from Western cultures. As a result, considering the role of cultural discrepancies in different factor structure findings (i.e., PID-5-BF and NEO), results from studies on the psychometrics of FFBI-SF in Western countries cannot be generalized to the Iranian population, and a separate study is needed to examine the factor structure, reliability, and validities of the FFBI-SF with Iranian samples.

In the current study, we examined the factor structure, reliability, and validity of the Persian version of the FFBI-SF with a sample of Iranian university students. First, confirmatory factor analysis (CFA) was conducted to test the proposed twelve-factor structure of the FFBI-SF [17]. Then, reliability indices values (Cronbach’s α and mean inter-item correlation values) were calculated to examine the reliability of the Persian FFBI-SF scores. We expected the Persian form of the FFBI-SF to demonstrate strong reliability indices values. Finally, the association of FFBI-SF subscales scores with other personality measures scores (e.g., Negative Affectivity, Antagonism, Disinhibition, and Consciousness) were calculated to examine the convergent/discriminant validities of the FFBI-SF scores. Specifically, we expected the FFBI-SF Total score to correlate positively to Negative Affective, Disinhibition, and Antagonism while negatively correlated with Consciousness and Agreeableness. Furthermore, we expected the FFBI-SF subscales to demonstrate convergent validity with their parent domain and discriminant validity with other personality domains (e.g., [17, 18, 23, 24, 43]).

## Methods

### Participants

Participants were 18-58 years old university students (*n* = 641, M-age = 28.04, *SD* = 8.21, 66.7% women) in Tehran who were recruited between April 2021 to July 2021.

### Procedure

The ethics committee of the Iran University of Medical Sciences first approved this study. Then, a demographic form (with three questions assessing age, gender, and education level), a forty-eight-item, twenty-five-item, and a twenty-item Likert online survey were developed. 641 participants were contacted through a secured online platform, and they provided online informed consent after reading the research purpose and being assured about the voluntary and confidential character of the study. Then they were asked to complete the questionnaires in the following order: The demographic form, FFBI-SF, PID-5-BF, and Mini-IPIP. Inclusion criteria consisted of being an undergraduate or graduate-level student, the age range of > 18, and having the interest to participate in the study.

### Measures

#### Five-factor borderline inventory - short form (FFBI-SF)

The FFBI-SF is a self-report measure that assesses BPD from the perspective of the FFM [17]. The FFBI-SF consists of 48 items which are rated on a 5-point Likert scale and yield one total score and 12 subscales scores. Chronbach’s α for the FFBI-SF Total score was .96 and .97 for student and Mturk samples, respectively. Further, the subscales have ranged from .68 (Oppositionality) to .92 (Dissociative Tendencies) for the student sample and .70 (Fragility and Oppositionality) to .92 (Dissociative Tendencies) in the Mturk sample [17].

#### Persian FFBI-SF

First, the questionnaire was obtained from the original developer (Hilary L. DeShong, Ph.D.). Then, it was translated from English to Persian by two translators skilled in both English and Persian. Next, Persian translations were matched and shared with another translator to back-translate items (i.e., from Persian to English). Afterward, the English translation was compared with the original text, and the back-translation was shared with the original developer. Finally, the measure was reviewed and revised based on the original developer’s comments. To examine the face validity of the measure, 30 students were recruited through the convenience sampling method and were asked to go through the questionnaire items and rate them with respect to suitability and clarity. Finally, for the content validity of the FFBI-SF, five Ph.D. degree specialists in clinical psychology were asked to rate the items concerning relevancy, clarity, and simplicity based on a 4-point Likert scale.

#### Personality inventory for DSM-5–brief form (PID-5-BF)

Krueger et al. [44] developed the PID-5-BF by extracting 25 items from the 220-item PID-5. PID-5-BF represents 21 of the 25 trait facets (facets not included: Restricted Affectivity, Rigid Perfectionism, Submissiveness, and Suspiciousness). Items of PID-5-BF are rated on a 4-point scale (0 = *very false or often false* to 3 = *very true or often true*), with higher scores representing greater dysfunction. Each of the five higher-order domains is represented by five items (Negative Affect: Items 8, 9, 10, 11, and 15; Detachment: Items 4, 13, 14, 16, and 18; Antagonism: Items 17, 19, 20, 22, and 25; Disinhibition: Items 1, 2, 3, 5, and 6; and Psychoticism: Items 7, 12, 21, 23, and 24). Elhami Athar and Ebrahimi [35] supported the five-factor model of the Iranian version of the PID-5-BF in the Iranian community and clinical samples and reported acceptable internal consistencies for the measure in both groups. Cronbach’s alpha and MICs for the PID-5-BF factors are shown in Table 1.

**Table 1:** Descriptive Statistics of the Modified FFBI-SF, PID-5-BF, and Mini IPIP (*n* = 641)

Measures	Mean (SD)	Skewness	Kurtosis	*α*	MIC
FFBI-SF					
FFBI-SF Total Score	114.10 (34.62)	.48	−.28	.95	.30
Anxious Uncertainty	11.78 (4.20)	−.05	−.86	.75	.43
Dysregulated Anger	11.75 (4.46)	.01	−.96	.82	.55
Despondence	9.32 (4.04)	.70	−.22	.75	.43
Self-Disturbance	9.63 (3.93)	.53	−.31	.70	.37
Behavioral Dysregulation	9.66 (3.92)	1.02	4.65	.64	.32
Affective Dysregulation	9.91 (3.82)	.36	−.64	.72	.40
Fragility	8.20 (3.53)	.80	.11	.68	.34
Dissociative Tendencies	7.90 (3.92)	.79	−.26	.78	.46
Distrustfulness	11.59 (3.92)	.10	−.73	.74	.42
Manipulativeness	7.86 (3.17)	.83	.48	.58	.27
Oppositional	6.86 (2.68)	.48	−.28	.54	.27
Rashness	9.59 (3.97)	.40	−.67	.77	.46
PID-5-BF					
Negative Affect	5.94 (3.78)	.27	−.71	.79	.43
Detachment	12.90 (4.59)	.26	−.66	.71	.33
Antagonism	3.96 (2.58)	.75	.86	.57	.22
Disinhibition	4.44 (3.41)	.60	−.13	.76	.39
Psychoticism	4.52 (3.41)	.44	−.67	.75	.38
Mini-IPIP					
Neuroticism	10.91 (3.86)	.23	−.51	.75	.43
Extraversion	12.82 (3.54)	−.12	−.33	.62	.29
Intellect/Imagination	14.83 (3.69)	−.53	−.41	.55	.23
Agreeableness	14.98 (2.79)	−.28	−.32	.49	.20
Conscientiousness	14.83 (3.69)	−.53	−.41	.73	.40

*Note*. *FFBI-SF* Five Factor Borderline Inventory - Short Form, *PID-5-BF* Personality Inventory for DSM-5-Brief Form, *Mini-IPIP* Mini International Personality Item Pool, *SD* Standard deviation, *α* Chrobach’s Alpha *MIC* mean interitem correlation

#### Mini international personality item Pool

The Mini-IPIP [45], a 20-item scale, is the short-form version of the 50-item International Personality Item Pool five-factor model [46]. Mini-IPIP includes five subscales, including neuroticism, extraversion, openness, agreeableness, and conscientiousness, with four items for each subscale. Items are rated on a 5-point Likert-type scale from 1 (*very inaccurate*) to 5 (*very accurate*). Each item is written as a statement, and participants rate how well it describes them. Sample items include “*Get upset easily*” (neuroticism), “*Am the life of the party*” (extraversion), “*Have a vivid imagination*” (openness to experience), “*Sympathize with others’ feelings*” (agreeableness), and “*Get chores done right away*” (conscientiousness). Cronbach’s alpha and MICs for the Mini-IPIP factors are shown in Table 1.

### Data analyses

We first calculated the descriptive information for all variables used in the present study, which are represented in Table 1. SPSS 20 was used to perform descriptive characteristics of the study sample and descriptive statistics of measures. The frequency table and box plots were implemented to identify and deal with outliers. The results indicated that the data was devoid of outliers; missing values were handled using the series mean method.

The literature on structural equation (e.g., [47]) has suggested that should preceding research confirm the factor structure of a measure, following validation studies should conduct confirmatory factor analysis (CFA) instead of exploratory factor analysis (EFA). Therefore, to test the proposed twelve-factor structure model of FFBI-SF, CFA was conducted through Lisrel 8.80 using the maximum likelihood estimator [48]. We examined the skewness and kurtosis statistics of each of the observable variables (i.e., measure’s items), and the results showed that all items were in the recommended skewness (− 3 to + 3) and kurtosis ranges (− 10 to + 10); therefore, the univariate normality was not violated (e.g., [49, 50]). Also, the relative multivariate kurtosis index as reported by the output from LISREL 8.80 was equaled to 1.23, which is less than 3, indicating that the data met the criteria of multivariate normality [51]. To examine model fit, we relied on the Tucker–Lewis index (TLI), the comparative fit index (CFI), the root mean square error of approximation (RMSEA), and standardized root mean squared residual (SRMR). We considered RMSEA and SRMR scores below .05 to indicate a good fit and scores between .05 and .08 indicating acceptable fit. Also, a TLI and CFI score of .95 or above was considered as excellent fit, and scores of .90 or more to indicate a good fit [52, 53]. A CFA was conducted to examine the twelve-factor model specified with the 48 items (observed variables) and twelve factors as latent and correlated constructs (4 items for each factor).

We also calculated the internal consistency of the FFBI-SF scores with Cronbach’s alpha (α), defined as low-to-marginal (≤ .59), marginal (.60 to .69), acceptable (.70 to .79), good (.80 to .89), and excellent (≥ .90) [54];. Considering the dependence of α on the number of items in a scale, we also computed mean inter-item correlation (MIC) as an additional indicator of the internal consistency, with values ranging from .15 to .50 being considered adequate [55].

Finally, to evaluate the convergent/discriminant validities of the interpretation of the FFBI-SF scores, Pearson correlation coefficients were examined between the FFBI-SF scores and other personality measures scores (i.e., Negative Affectivity, Disinhibition, Antagonism, Consciousness, and Agreeableness). We hypothesized that the FFBI-SF Total and subscales scores demonstrate moderate to strong positive correlations with PID-5-BF subscales scores and with Mini-IPIP Neuroticism subscale, while they yield low to moderate negative associations with Mini-IPIP Extraversion, Intellect/Imagination, Agreeableness, and Conscientiousness subscales. More specifically, we expected the Dissociative Tendencies subscale yield its hights correlation with PID-5-BF’s Psychoticism. Similarly, we assumed the Manipulativeness and Oppositional scores yield strong significant associations with Antagonism; and Rashness yields its highest positive and negative correlations with Disinhibition and Conscientiousness, respectively. Correlation coefficients were interpreted as ≤ .30 = small; .30-.50 = medium; and ≥ .50 = strong effect sizes [56].

## Results

### Confirmatory factor analysis

The results of the CFA indicated that the twelve-factor model of the FFBI-SF reached acceptable (RMSEA = .07, SRMR = .061) and excellent (CFI = .96, TLI = .96) fit. All loadings were significant and higher than the threshold loading (i.e., < .30) [57] except for item 47, which had a loading of .12 (Table 2). Thus, we conducted a CFA without item 47, which resulted in acceptable (RMSEA = .07, SRMR = .060) and excellent (CFI = .96, TLI = .96) model fit.

**Table 2:** FFBI-SF Items Loadings

Subscales													
Item Number	Item content	AU	DA	DES	SD	BD	AD	F	DT	DIS	MA	OPP	RA
1	I tend to be quite anxious.	.60 ^*^											
13	I worry a great deal.	.76 ^*^											
25	I worry a lot about people leaving me.	.61^*^											
37	I worry a lot about things that are out of my control.	.69 ^*^											
2	I have had quite a few angry outbursts.		.67 ^*^										
14	My anger often feels out of control.		.77 ^*^										
26	My anger at times gets the better of me.		.78 ^*^										
38	My anger has at times gotten me into trouble.		.75 ^*^										
3	I sometimes feel worthless.			.65 ^*^									
15	I have thought about ways to kill myself.			.60 ^*^									
27	I often feel sad.			.76 ^*^									
39	I have thought about suicide since I was a teenager.			.55 ^*^									
4	I can be so different with different people that it’s like I’m not the same person.				.38 ^*^								
16	I can be so different with different people that I wonder who I am.				.57 ^*^								
28	I tend to feel like I don’t belong with anyone.				.68 ^*^								
40	I often feel like an outcast.				.72 ^*^								
5	I frequently have urges to do things that get me into trouble.					.43 ^*^							
17	Sometimes I let myself get swept away by my urges.					.59 ^*^							
29	When I am upset, I often do things that later cause me problems.					.49 ^*^							
41	I have done a lot of things impulsively that I later regret.					.72 ^*^							
6	My emotions can spiral out of control.						.47 ^*^						
18	I don’t seem to have much control over how I feel.						.66 ^*^						
30	My mood shifts rapidly from one feeling to another.						.69 ^*^						
42	I have a difficult time controlling my mood.						.71 ^*^						
7	Harming myself is one of the few ways I can tolerate my emotions.							.47 ^*^					
19	I have threatened to commit suicide.							.51 ^*^					
31	Even minor setbacks can cause a great deal of drama in my life.							.65 ^*^					
43	I don’t think I can continue to live like this							.70 ^*^					
8	I have felt that things were unreal and I was detached from life.								.69 ^*^				
20	Sometimes I feel like I am no longer connected to my body.								.50 ^*^				
32	I sometimes feel like I am not real.								.78 ^*^				
44	I sometimes feel that nothing is real.								.77 ^*^				
9	I am often distrustful of other people.									.70 ^*^			
21	It’s really hard for me to trust people.									.72 ^*^			
33	People are not as loyal to me as I wish they were.									.60 ^*^			
45	I have not been able to trust some of my closest friends.									.70 ^*^			
10	I sometimes do things I shouldn’t to get people to do things I want or need.										.55 ^*^		
22	Other people have called me manipulative.										.55 ^*^		
34	I have been known to massage the truth to get my way.										.54 ^*^		
46	At times you have to be dishonest and manipulative to get what you need.										.45 ^*^		
11	I tend to get into lots of arguments.											.43 ^*^	
23	I will make threats to get people to do things.											.49 ^*^	
35	I often get into arguments with people who are close to me.											.64 ^*^	
47	I am easy to get along with.											.12 ^*^	
12	I get into trouble because I don’t think things through.												.66 ^*^
24	I tend to act quickly without thinking things through.												.72 ^*^
36	Others have said that I do not think before I act.												.74 ^*^
48	I’ve done some pretty bad things on impulse.												.60 ^*^

*Note*. *FFBI-SF-SF* Five Factor Borderline Inventory - Short Form, *AU* Anxious Uncertainty, *DA* Dysregulated Anger, *DES* Despondence, *SD* Self-Disturbance, *BD* Behavioral Dysregulation, *AD* Affective Dysregulation, *F* Fragility, *DT* Dissociative Tendencies, *DIS* Distrustfulness, *MA* Manipulativeness, *OPP* Oppositional, *RA* Rashness; * = All paths are significant *p* < .05

### Internal consistency and correlations between the FFBI-SF scores

As shown in Table 1, when relying on Cronbach’s alpha (*α*) as the index of internal consistency, the reliability of FFBI-SF scores ranged from unacceptable (Oppositional; *α* = .54) to excellent ranges (FFBI-SF Total Score; *α* = .95), and the median *α* for the FFBI-SF scores was .76. On the other hand, when we examine the internal consistency of the FFBI-SF scores based on MIC values, which is the straightforward measure of the internal consistency, the FFBI-SF Total and subscales scores demonstrated adequate internal consistency, though the MIC value of Dysregulated Anger subscale was higher than the recommended range. Significant zero-order correlations were also found between FFBI-SF subscale scores and the FFBI-SF Total score and between the twelve FFBI-SF subscales scores (see Table 1).

### Convergent/discriminant validity

FFBI-SF Total and subscale scores were significantly associated with other related personality measures scores, i.e., PID-5-BF and Mini IPIP. The FFBI Total score had a strong positive correlation with Negative Affect, Detachment, Antagonism, Disinhibition, Psychoticism, and Neuroticism (*r’s* = .51-.85), while it was negatively associated with Extraversion, Conscientiousness, Agreeableness and Intellect/Imagination (*r’s* = −.18 to −.37). Furthermore, small to moderate and strong significant positive correlations were observed between FFBI-SF subscale scores and PID-5-BF and Mini-IPIP subscale scores. For instance, Anxious Uncertainty, Dysregulated Anger, Despondence, Affective Dysregulation, Self-Disturbance, Behavioral Dysregulation, and Fragility scores had strong and positive correlations with Negative Affect, Detachment, Antagonism, Disinhibition, Psychoticism, and Neuroticism (*r’s* = .30 - .82), while they had small to moderate significant negative associations with Extraversion, Conscientiousness, Intellect/Imagination, and Agreeableness (*r’s* = −.08 to −.35) (For more information, see Table 3).

**Table 3:** Pearson correlation between the Modified FFBI-SF, PID-5-BF, and Mini IPIP, and intercorrelations among FFBI-SF subscales (*n* = 641)

Measures	FFBI-Total Score	AU	DA	DES	SD	BD	AD	F	DT	DIS	MA	OPP	RA
**FFBI-SF**													
FFBI-SF Total Score	–												
Anxious Uncertainty	.74^**^	–											
Dysregulated Anger	.79^**^	.60^**^	–										
Despondence	.75^**^	.60^**^	.53^**^	–									
Self-Disturbance	.81^**^	.56^**^	.57^**^	.65^**^	–								
Behavioral Dysregulation	.78^**^	.50^**^	.60^**^	.47^**^	.55^**^	–							
Affective Dysregulation	.85^**^	.58^**^	.70^**^	.60^**^	.64^**^	.68^**^	–						
Fragility	.83^**^	.59^**^	.59^**^	.72^**^	.67^**^	.59^**^	.68^**^	–					
Dissociative Tendencies	.71^**^	.44^**^	.40^**^	.54^**^	.60^**^	.48^**^	.53^**^	.61^**^	–				
Distrustfulness	.69^**^	.54^**^	.47^**^	.46^**^	.57^**^	.48^**^	.51^**^	.45^**^	.48^**^	–			
Manipulativeness	.69^**^	.38^**^	.44^**^	.42^**^	.57^**^	.50^**^	.52^**^	.56^**^	.50^**^	.46^**^	–		
Oppositional	.69^**^	.40^**^	.59^**^	.39^**^	.47^**^	.56^**^	.57^**^	.53^**^	.39^**^	.42^**^	.51^**^	–	
Rashness	.76^**^	.49^**^	.59^**^	.41^**^	.48^**^	.69^**^	.67^**^	.56^**^	.46^**^	.46^**^	.50^**^	.57^**^	–
**PID-5-BF**													
Negative Affect	.68^**^	.68^**^	.54^**^	.52^**^	.49^**^	.52^**^	.59^**^	.56^**^	.40^**^	.49^**^	.37^**^	.41^**^	.53^**^
Detachment	.85^**^	.70^**^	.72^**^	.64^**^	.82^**^	.61^**^	.76^**^	.67^**^	.55^**^	.53^**^	.54^**^	.55^**^	.56^**^
Antagonism	.51^**^	.30^**^	.38^**^	.33^**^	.40^**^	.41^**^	.41^**^	.41^**^	.35^**^	.34^**^	.57^**^	.41^**^	.39^**^
Disinhibition	.64^**^	.43^**^	.47^**^	.45^**^	.45^**^	.56^**^	.59^**^	.51^**^	.44^**^	.36^**^	.45^**^	.43^**^	.66^**^
Psychoticism	.64^**^	.41^**^	.40^**^	.45^**^	.57^**^	.49^**^	.52^**^	.53^**^	.62^**^	.49^**^	.46^**^	.40^**^	.49^**^
**Mini-IPIP**													
Neuroticism	.74^**^	.63^**^	.61^**^	.63^**^	.59^**^	.52^**^	.67^**^	.64^**^	.47^**^	.45^**^	.43^**^	.48^**^	.55^**^
Extraversion	−.18^**^	−.12^**^	−.08^*^	−.22^**^	−.28^**^	−.07	−.13^**^	−.14^**^	−.20^**^	−.24^**^	−.12^**^	.08	−.03
Conscientiousness	−.37^**^	−.20^**^	−.25^**^	−.31^**^	−.27^**^	−.34^**^	−.35^**^	−.32^**^	−.27^**^	−.15^**^	−.26^**^	−.22^**^	−.34^**^
Intellect**/**Imagination	−.26^**^	−.17^**^	−.14^**^	−.21^**^	−.15^**^	−.20^**^	−.22^**^	−.23^**^	−.20^**^	−.18^**^	−.22^**^	−.15^**^	−.27^**^
Agreeableness	−.24^**^	−.03	.07	−.18^**^	−.27^**^	−.17^**^	−.17^**^	−.20^**^	−.29^**^	−.21^**^	−.28^**^	−.11^**^	−.15^**^

*Note*. *FFBI-SF –SF* Five Factor Borderline Inventory - Short Form, *PID-5-BF* Personality Inventory for DSM-5-Brief Form, *Mini-IPIP* Mini International Personality Item Pool, *AU* Anxious Uncertainty, *DA* Dysregulated Anger, *DES* Despondence, *SD* Self-Disturbance, *BD* Behavioral Dysregulation, *AD* Affective Dysregulation, *F* Fragility, *DT* Dissociative Tendencies, *DIS* Distrustfulness, *MA* Manipulativeness; *OPP* = Oppositional; *RA* = Rashness; *p < .05; **p < .001

## Discussion

In the current study, we aimed to examine the factor structure, reliability, and convergent/discriminant validities of FFBI-SF with a sample of 643 Iranian university students. Our first aim was to test the proposed twelve-factor structure of the FFBI-SF [17]. The confirmatory factor analysis results indicated that the originally proposed twelve-factor structure model of the FFBI-SF reached an adequate to excellent fit. However, as shown in Table 2, all of the items reached the minimum loading threshold of <.30 [57], item 47 (*“I am easy to get along with”*), had a very low loading (.12). Thus, we removed this item from the model, and this modified twelve-factor model yielded an adequate to excellent fit. The low loading of item 47 might stem from the fact that it was a reversed item (e.g., [58]). Also, the poor function of this item might be related to the tendency of respondents to answer questions in a manner that is viewed favorably by others (i.e., social desirability), which is reinforced by Iranian collectivistic culture. This item does not seem to be necessary (especially in Iranian collectivistic culture) and it reduces the validity of the measure (at least for OPP subscale). Therefore, we conducted the rest of the analyses using the modified twelve-factor model.

Conceptually, the FFBI-SF subscale scores should measure interrelated aspects of a unique overarching construct of Borderline Personality Disorder. Accordingly, it is expected that the subscales should demonstrate moderate to strong associations with each other. Consistent with such hypothesis and previous findings for the English version of the FFBI and FFBI-SF (e.g., [17, 18]), our results indicated significant zero-order correlations between FFBI-SF subscale scores with the FFBI-SF Total score and between the twelve FFBI-SF subscales scores.

Furthermore, our results indicated that the Cronbach’s alpha (*α*) for the FFBI-SF scores ranged from unacceptable (Oppositional) to excellent (FFBI-SF Total score) ranges. However, when we relied on MIC values as the measure of the internal consistency, the FFBI-SF Total and subscale scores demonstrated adequate internal consistency [55], though the Dysregulated Anger subscale had a MIC value of higher than the recommended range, indicating that the subscale’s items are correlated to a greater extent, and they may be repetitive in measuring the intended construct. Since Cronbach’s alpha is a function of the number of items, it is not a precise index of internal consistency, especially in the current study in which the subscales include few items (i.e., only four items for each subscale). The degree of interitem correlation (MIC) is not relevant to the number of items and provides a direct indication of the internal consistency [55, 59, 60]. Therefore, it can be concluded that the Persian FFBI-SF Total and subscales scores are internally consistent.

The current study also examined associations between FFBI-SF scores and other personality measures (i.e., PID-5-BF and Mini-IPIP) to support the convergent/discriminant validity of the Persian version of this measure. Consistent with prior studies [17, 18, 23, 24, 43], our results indicated that the FFBI Total score was strongly and positively associated with Negative Affect, Detachment, Antagonism, Disinhibition, Psychoticism, and Neuroticism, while it had significant negative correlations with Extraversion, Conscientiousness, Agreeableness, and Intellect/Imagination. Furthermore, with respect to FFBI-SF subscales score, our results indicated that Anxious Uncertainty, Dysregulated Anger, Despondence, Affective Dysregulation, Self-Disturbance, Behavioral Dysregulation, and Fragility scores had strong positive correlations with Negative Affect, Detachment, and Neuroticism, while they demonstrated small to moderate significant positive correlations with Antagonism, Disinhibition, and Psychoticism and significant negative associations with Extraversion, Conscientiousness, Intellect/Imagination, and Agreeableness. As expected, Dissociative Tendencies had its highest correlation with Psychoticism. In the same vein, Rashness had its highest positive and negative correlations with Disinhibition and Conscientiousness, respectively. Additionally, the Manipulativeness and Oppositional scores had moderate significant correlations with the Negative Affectivity and Neuroticism and a strong significant association with Antagonism compared to other FFBI-SF subscales (e.g., Anxious Uncertainty, Dysregulated Anger, Despondence, and Behavioral Dysregulation); simultaneously, they had significant negative associations with the Conscientiousness and Agreeableness subscale scores.

While there was good convergent validity, the discriminant validity was not as clear. For instance, all FFBI-SF subscales had strong significant associations with Detachment. This could have occurred for several reasons. First, three of the subtraits within Detachment (i.e., depressivity, suspiciousness, and restricted affectivity) are also traits within the domain Negative Affectivity, indicating the potential for conceptual overlap between these two domains specifically – one of which is at the core of BPD (i.e., negative affectivity). Alternatively, it could be that these results stem from the interpersonal disturbance, which is among the hallmarks of BPD, and that those with BPD adopt withdrawal behavior or social detachment and limit interpersonal relationships to protect themselves against abandonment altogether [61]; thus, it might be expected that BPD scores as measured by FFBI-SF, be strongly associated with Detachment. A third plausible explanation may be that one of the measures used in the current study potentially has discriminant issues. In sum, the results provide support for the convergent validity of the interpretation of the FFBI-SF Total and subscale scores in an Iranian university student sample. However, more research is needed to assess if the discriminant validity concerns are related solely to the PID-5 or to other personality and pathological personality measures.

Our findings should be interpreted with respect to a few limitations. First, for data gathering, we solely relied on self-report data to examine the convergent/discriminant validity. Therefore, associations of self-report BPD measure with PID-5-BF and Mini-IPIP scores may partly be explained by shared method variance. Second, in the current study, the study sample included a non-clinical university student sample whose diagnostic status was not assessed. Third, our study sample included only university students sample, so our findings should not be generalized to other groups. Future studies could extend the results of the present study by including clinical samples, especially patients with personality disorders and comparing the results with a large community sample. Finally, we only tested the original 12-factor model of the FFBI-SF, but there might be other superior models for the Persian FFBI-SF, which are untested here.

In conclusion, this is the first study to translate and provide initial validation on a Persian version of the dimensional trait measure the FFBI-SF [17]. This measure offers a brief (5-10 min) but a wholistic assessment of the traits of BPD, providing both a total BPD score and scores of the underlying facet-level traits of BPD. This could be useful for clinicians in tracking reported traits of BPD at the start of and across treatment. Additionally, the brevity allows the FFBI-SF to be useful as part of a large battery of measures in a research context. Future studies should continue to include the Persian version of the FFBI-SF in addition to interview and informant reports of personality and personality disorders in both clinical and more generalizable samples, in addition to including measures of other criteria to provide further construct and criterion validity of the measure.

## Data Availability

The datasets generated during and/or analyzed during the current study are available from the corresponding author on reasonable request.
